# Treatment optimisation for hepatitis C in the era of combination direct-acting antiviral therapy: a systematic review and meta-analysis

**DOI:** 10.12688/wellcomeopenres.15411.1

**Published:** 2019-09-06

**Authors:** Christopher R. Jones, Barnaby F. Flower, Ella Barber, Bryony Simmons, Graham S. Cooke

**Affiliations:** 1 Department of Infectious Disease, Imperial College London, London, W2 1NY, UK; 2 Oxford University Clinical Research Unit, Centre for Tropical Medicine, Ho Chi Minh City, Vietnam

**Keywords:** hepatitis C virus, stratified medicine, personalised medicine, treatment optimisation, direct acting antivirals, sustained virologic response, systematic review

## Abstract

**Background:** Prior to direct-acting antiviral (DAA) therapy, personalised medicine played an important role in the treatment of hepatitis C virus (HCV). Whilst simplified treatment strategies are central to treatment scale-up, some patients will benefit from treatment optimisation. This systematic review and meta-analysis explores treatment optimisation strategies in the DAA era.

**Methods:** We systematically searched Medline, Embase, and Web of Science for studies that adopted a stratified or personalised strategy using a licensed combination DAA regimen, alone or with additional agents. We performed a thematic analysis to classify optimisation strategies and a meta-analysis of sustained virologic response rates (SVR), exploring heterogeneity with subgroup analyses and meta-regression.

**Results:** We included 64 studies (9450 participants). Thematic analysis found evidence of three approaches: duration, combination, and/or dose optimisation. We separated strategies into those aiming to maintain SVR in the absence of predictors of failure, and those aiming to improve SVR in the presence of predictors of failure. Shortened duration regimens achieve pooled SVR rates of 94.2% (92.3-95.9%) for 8 weeks, 81.1% (75.1-86.6%) for 6 weeks, and 63.1% (39.9-83.7%) for ≤4 weeks. Personalised strategies (100% vs 87.6%; p<0.001) and therapy shortened according to ≥3 host/viral factors (92.9% vs 81.4% or 87.2% for 1 or 2 host/viral factors, respectively; p=0.008) offer higher SVR rates when shortening therapy. Hard-to-treat HCV genotype 3 patients suffer lower SVR rates despite treatment optimisation (92.6% vs 98.2%; p=0.001).

**Conclusions:** Treatment optimisation for individuals with multiple predictors of treatment failure can offer high SVR rates. More evidence is needed to identify with confidence those individuals in whom SVR can be achieved with shortened duration treatment.

## Introduction

Viral hepatitis is a leading cause of mortality globally ^1^ and an estimated 71 million individuals are infected with hepatitis C virus (HCV) ^2^. The recent transformation in HCV treatment has led to ambitious World Health Organization (WHO) targets for its elimination as a public health threat by 2030; ^3^ meeting those targets will require at least 80% of infected individuals accessing treatment and in richer countries the ambition will be to exceed this.

Simplified treatment strategies will be essential for rapid treatment scale-up ^4^. However, some subgroups experience worse cure rates with standard therapies and may benefit from a stratified or personalised approach. Stratified medicine refers to the separation of patients into subgroups based on factors known to be associated with outcome. Personalised medicine goes further, individualising therapy according to the unique host and/or viral context. Both are used for treatment optimisation. Such approaches may become increasingly relevant for patients who struggle to engage with care, and in some settings may be more cost-effective ^5^. The balance between simplified and personalised care has been well described for tuberculosis ^6^. An understanding of the risks and benefits of a stratified or personalised approach for HCV is required to inform treatment in different settings.

Treatment strategies centred on direct acting antiviral (DAA) combinations are evolving rapidly, taking into account new knowledge of efficacy, safety, simplicity, and cost. Recent guidelines advocate treatment optimisation for certain subgroups ^7, 8^. Both host (liver disease stage, treatment history) and viral (HCV genotype, baseline viral load) factors inform treatment decisions, with the duration and/or regimen being adapted to optimise sustained virologic response rates (SVR).

We have carried out the first detailed review of treatment optimisation strategies in the DAA era. We used a mixed methods approach to synthesise data on such strategies for the management of HCV within a theoretical framework. We aimed to initiate a discussion on the role of treatment optimisation within an increasingly simplified treatment landscape.

## Methods

### Search strategy

We performed this review in accordance with PRISMA 2009 guidelines ^9^. We searched Medline, Embase, and Web of Science using a free text strategy ( *Extended data*, Supplementary Table 1 ^10^) combining hepatitis C; host/viral factors associated with treatment outcome; and DAAs used in combination regimens. The search period spans January 2013 (year of first publication of phase 2 trial of sofosbuvir) and July 2019 (end of review period). We searched the National Institutes of Health clinical trials registry ^11^ and the European (EASL) or American (AASLD) International Liver Congress websites to identify ongoing trials. We reviewed reference lists to identify additional studies.

### Study selection criteria

One reviewer (CRJ) screened abstracts against pre-determined inclusion/exclusion criteria. A second reviewer (BFF) independently screened a 20% sample of all records. We resolved discrepancies through discussion between reviewers (CRJ, BFF) and the senior author (GSC). We included phase 2–4 interventional and observational studies of adults (>18 years old) with chronic HCV (>6 months duration) where SVR 12 weeks post-therapy was the primary outcome and at least one treatment arm met our definition of a stratified or personalised treatment strategy. We only included studies that assessed a licensed combination DAA regimen, alone or in combination with other DAAs, interferon (IFN), or ribavirin (RBV). We excluded studies assessing one DAA in combination with IFN or RBV, or unlicensed DAAs (unless used alongside a licensed regimen).

### Stratified or personalised treatment strategy definition

Evidence of *a priori* stratification of patients into different treatment approaches based on predictors of response to therapy ^12– 14^. Strategies were considered stratified if patients were separated into broad subgroups according to prognostic factors, e.g. treatment history, liver disease stage, baseline HCV viral load. Strategies were considered personalised if therapy was individualised e.g. viral resistance testing, on treatment response kinetics, host genetics. Included strategies contained an adaptation to standard treatment, either defined in guidelines ^7, 8^ or by an equivalent 12 week standard of care. *A priori* or *post hoc* stratification for analysis only is not included in this definition.

### Data extraction

The first reviewer extracted data into a pre-designed Excel spreadsheet v16 (Microsoft Excel, RRID:SCR_016137). Where trials included multiple treatment arms, we only extracted data from arms that used a stratified or personalised strategy. We identified the following data *a priori* for extraction: year; author; journal; title; location; design; population; sample size; DAA regimen(s); treatment optimisation approach; host and/or viral optimisation factors; baseline demographics; resistance data at point of treatment failure; and SVR rates from intention-to-treat and per-protocol analyses.

### Quality assessment

We assessed included studies for methodological quality using the Cochrane Risk of Bias tool for randomised trials ^15^ within Review Manager v5.3 (RevMan, RRID:SCR_003581), or the Newcastle Ottawa Scale for non-randomised studies ^16^. Since most trials were non-comparative, we modified the Newcastle Ottawa Scale by removing the comparability domain. We only considered virologic outcome. Since SVR is a highly objective outcome measure, we did not consider lack of blinding a threat to validity.

### Thematic analysis

To characterise treatment strategies and guide quantitative analyses, we performed a thematic analysis as previously described ^17^. We examined the methods of each study in detail using a deductive theory-driven approach, coding data related to treatment strategy and predictive factors. We analysed codes in order to identify themes. We then re-analysed the data using an inductive approach, reviewing and refining themes before generating a conceptual framework.

### Quantitative analysis

We calculated pooled SVR rates using a DerSimonian and Laird random effects model ^18^ with the Freeman-Tukey double arcsine transformation to stabilise variances ^19^. We weighted effect sizes using an inverse variance approach and calculated 95% confidence intervals using the Clopper-Pearson exact method ^20^. We assessed statistical heterogeneity using Higgin’s I ^2^ test (0–25%, 25–75%, and >75% representing low, moderate, and high degrees of inter-study heterogeneity, respectively) ^21^.

We conducted pre-defined subgroup analyses to explore clinical variables associated with SVR (stage of liver disease, treatment history, genotype 3) or strategic variables that we hypothesised are associated with SVR in the context of treatment optimisation (duration, RBV use, number of host/viral treatment optimisation factors, pangenotypic vs genotype-specific regimen, stratified vs personalised approach). We performed a random effects meta-regression to explore clinical (stage of liver disease, treatment history, genotype 3, mean age, male proportion, mean body mass index (BMI), baseline HCV RNA, IL28B CC proportion) and methodological (duration, RBV use, number of host/viral treatment optimisation factors, randomisation, sample size, trial design, pangenotypic vs genotype-specific regimen, stratified vs personalised approach) sources of heterogeneity. We constructed a multivariable model using variables identified as significant (p≤0.1) in the univariable analysis. For studies not reporting the mean of continuous variables, we estimated mean and variance using the median and range ^22^. We did not formally assess publication bias since funnel plots are inaccurate in meta-analysis of proportion studies ^23^. We performed all analyses in Stata v14.1 (Stata, RRID:SCR_012763) using the metaprop ^24^ and metareg ^25^ commands. We considered all p values as significant if p≤0.05, unless otherwise stated.

To explore the prevalence of resistance-associated substitutions (RASs) at the point of virologic failure following a shortened treatment duration, we performed a separate pooled analysis of virologic failure resistance data, where available. We considered resistance to all three classes of DAAs, and both treatment-enriched and treatment-emergent substitutions. We stratified data by treatment duration and performed the analysis using a Chi-square test for trend in Prism v7 (GraphPad Prism, RRID:SCR_002798).

### Role of the funding source

There was no funding source for this study. The corresponding author had full access to all the data in the study and had final responsibility for the decision to submit for publication.

## Results

### Thematic analysis

Treatment optimisation strategies were categorised into three approaches: i) duration optimisation – shorten or lengthen therapy, with or without a response-guided element; ii) combination optimisation – add or remove DAAs, RBV, or IFN, or select a regimen with an advantageous characteristic e.g. higher genetic resistance barrier; and iii) dose optimisation – adjust dose or dosing schedule, either by increasing frequency or intermittent dosing. Personalised treatment strategies draw on ≥1 of these approaches ( *Extended data*, Supplementary Figure 1 ^10^). Host factors used to optimise treatment were stage of liver disease, treatment history, and weight/BMI. We did not find any studies using sex, ethnicity, or IL28B genotype. Viral factors were HCV genotype, baseline viral load, resistance-associated substitutions (RAS), and viral kinetics.

The intended purpose of treatment optimisation allows distinction of two groups: i) those with factors known to be negative predictors of SVR, in whom treatment optimisation aims to improve SVR rates compared to those expected with standard care – ‘improve SVR’ group; and ii) those without negative predictors, in whom the intention is to simplify/shorten therapy whilst achieving SVR rates non-inferior to those expected with standard care – ‘maintain SVR’ group.

### Summary of studies

**Figure 1.**
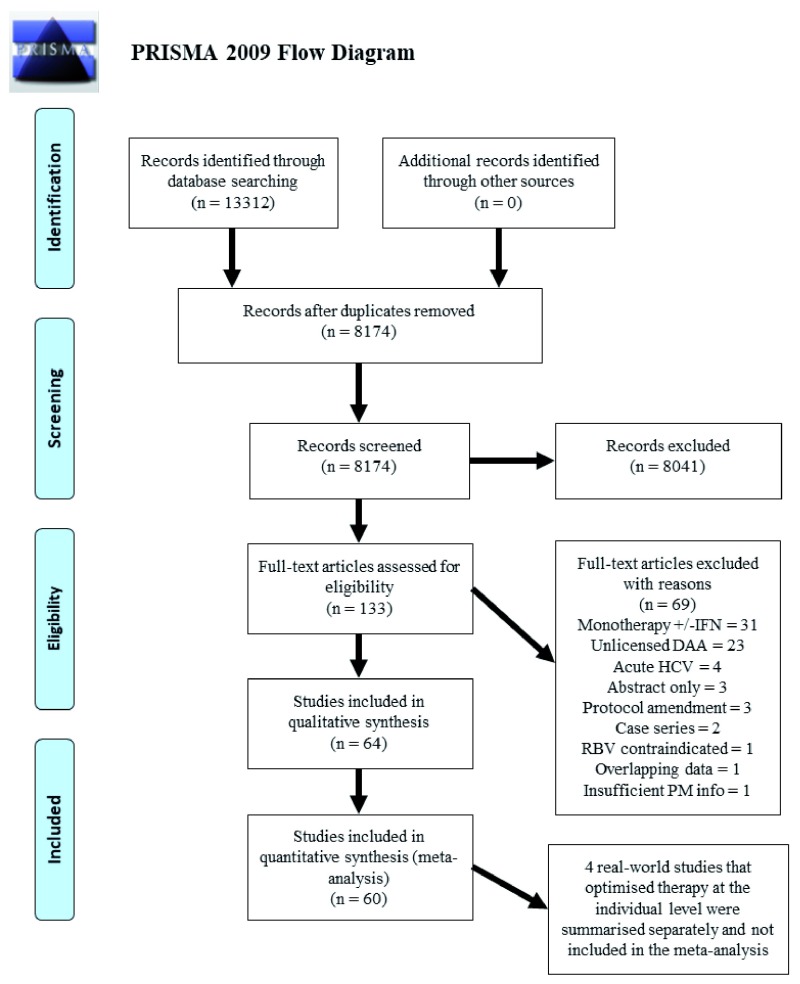
PRISMA flow diagram. Abbreviations: IFN - interferon; DAA - direct-acting antiviral; HCV - hepatitis C virus; RBV - ribavirin; PM - personalised medicine.

We identified 133 studies for full text review and 64 studies for final inclusion ( Figure 1) ^26– 89^. We extracted data from 104 treatment arms including 9450 participants ( Table 1). We identified six (9.4%) randomised controlled trials (RCT). Three (4.7%) RCTs were designed and powered to test an optimised treatment strategy, demonstrating non-inferiority to a standard regimen ^35, 57, 88^. Most treatment arms were quasi-experimental (74 arms – 71.2%) and included <50 participants (72 arms – 69.2%). Adjustment to the duration and combination of therapy was the most common optimisation strategy (52 arms – 50%), followed by duration (39 arms – 37.5%). Only one (1%) treatment arm adjusted the dosing schedule ^83^.

**Table 1.** Summary of overall study characteristics. Host and viral factors refer to the factors considered when optimising treatment strategies.

	Studies, n (%)	Arms, n (%)	Patients, n (%)
Total	64 (100)	104 (100)	9450 (100)
**Study design**			
Randomised controlled trial	6 (9.4)	8 (7.7)	1025 (10.8)
Quasi-experimental	40 (62.5)	74 (71.2)	2521 (26.7)
Observational	18 (28.1)	22 (21.2)	5904 (62.5)
**DAA regimen (+/- RBV)**			
Pangenotypic	19 (29.7)	37 (35.6)	1664 (17.6)
SOF/VEL/VOX	4 (6.3)	15 (14.4)	724 (7.7)
GLE/PIB	8 (12.5)	10 (9.6)	486 (5.1)
SOF/DCV	5 (7.8)	7 (6.7)	273 (2.9)
SOF/VEL	2 (3.1)	5 (4.8)	181 (1.9)
Genotype-specific	45 (70.3)	67 (64.4)	7786 (82.4)
SOF/LDV	24 (37.5)	32 (30.8)	6210 (65.7)
OMV/PTV/r +/- DSV	5 (7.8)	6 (5.8)	488 (5.2)
SOF/GZV/EVR	3 (4.7)	9 (8.7)	184 (1.9)
Other	13 (20.3)	20 (19.2)	904 (9.6)
**Number of subjects per arm**			
<50	36 (56.3)	72 (69.2)	1675 (17.7)
≥50	28 (43.8)	32 (30.8)	7775 (82.3)
Optimised treatment strategy *			
Duration optimisation	..	39 (37.5)	6576 (69.6)
Combination optimisation	..	8 (7.7)	424 (4.5)
Duration + combination optimisation	..	52 (50)	1881 (19.9)
Combination + dosing schedule optimisation	..	1 (1)	6 (0.1)
Case-by-case individualisation	..	4 (3.8)	563 (6)
Response-guided therapy	..	4 (3.8)	78 (0.8)
Host factors *			
HCV treatment history	..	86 (82.7)	8432 (89.2)
Liver disease stage	..	81 (77.9)	8225 (87)
Age	..	2 (1.9)	32 (0.3)
Weight	..	2 (1.9)	32 (0.3)
Viral factors *			
Baseline viral load	..	14 (13.5)	4338 (45.9)
RAS testing	..	8 (7.7)	724 (7.7)
Viral kinetics	..	5 (4.8)	106 (1.1)
Number of factors used *			
1	..	32 (30.8)	1976 (20.9)
2	..	55 (52.9)	2704 (28.6)
≥3	..	17 (16.3)	4770 (50.5)
Theme of optimisation *			
Maintain SVR in absence of negative predictors	..	63 (60.6)	6847 (72.5)
Improve SVR in presence of negative predictors	..	37 (35.6)	2040 (21.6)
Both	..	4 (3.8)	563 (6)
**Optimisation strategy**			
Stratified	54 (84.4)	91 (87.5)	8620 (91.2)
Personalised	10 (15.6)	13 (12.5)	830 (8.8)

*Number of studies not provided - some include multiple treatment arms that use different strategies. Abbreviations: DAA - direct acting antiviral; RBV - ribavirin; SOF - Sofosbuvir; LDV - Ledipasvir; VEL - Velpatasvir; VOX - Voxilaprevir; OMV - Ombitasvir; PTV/r - Paritaprevir/ritonavir; DSV - Dasabuvir; GZV - Grazoprevir; RZV - Ruzasvir; UFV - Uprifosbuvir; DCV - Daclatasvir; GLE - Glecaprevir; PIB - Pibrentasvir; IFN - interferon; BD - twice daily.

In total, 63 arms (60.6%) fall within the ‘maintain SVR’ group and 37 arms (35.6%) within the ‘improve SVR’ group. Four (3.8%) real-world observational studies personalised treatment on a case-by-case basis. Most arms used treatment history (82.7%) and/or stage of liver disease (77.9%) when optimising therapy. Eight arms (7.7%) personalised according to baseline resistance testing. Individual studies are summarised in the *Extended data*, Supplementary Table 2 ^10^.

Treatment optimisation strategies within the ‘maintain SVR’ and ‘improve SVR’ groups used opposing strategies to study participants with different prognostic characteristics. Consequently, we anticipated a difference in treatment outcome and performed separate meta-analyses. A narrative summary of the four real-world studies that personalised therapy on a case-by-case basis is provided separately.

### Maintain SVR group meta-analysis

**Figure 2.**
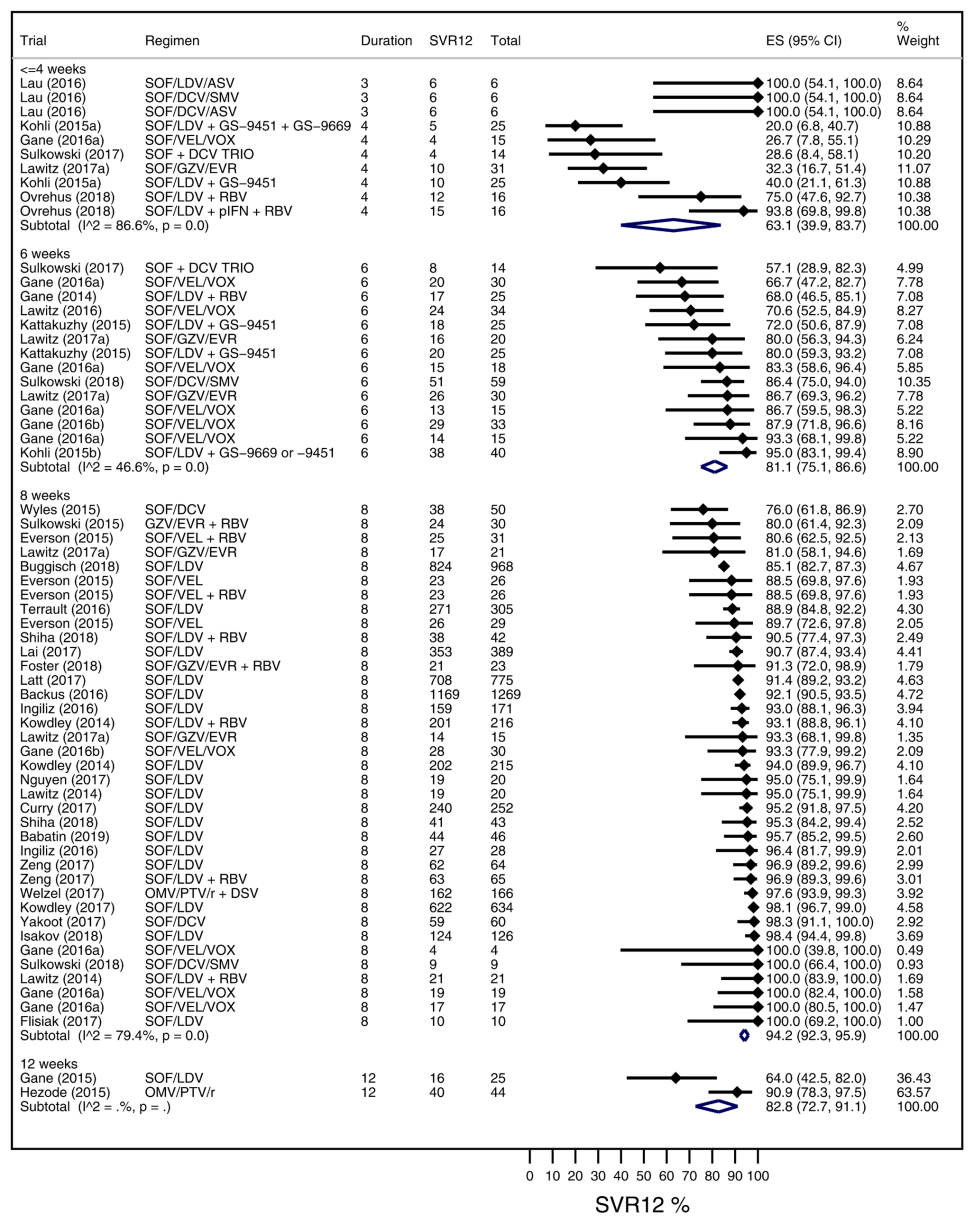
A Forest plot displaying pooled intention-to-treat SVR rates for optimised treatment arms in the maintain SVR group stratified by treatment duration.

Figure 2 displays pooled intention-to-treat SVR rates for strategies that aim to maintain SVR in the absence of negative predictors. Pooled SVR for regimens of ≤4 weeks duration was 63.1% (39.9-83.7%; I ^2^ = 86.6%), 6 weeks duration was 81.1% (75.1-86.6%; I ^2^ = 46.6%), and 8 weeks duration was 94.2% (92.3-95.9%; I ^2^ = 79.4%). There was significant intra- and inter-group heterogeneity (p=<0.001). A *post hoc* sensitivity analysis excluded arms that shorten therapy in the presence of negative predictors of SVR, which did not alter pooled estimates (data not shown).

Subgroup analysis ( Table 2) found higher SVR rates for strategies that used ≥3 personalised treatment factors (92.9%; 95% CI 90.4-95.1%) compared to one (81.4%; 95% CI, 71.1-90%) or two (87.2%; 95% CI, 82.1-91.6%). The test for subgroup differences suggests that this effect is significant (p=0.008), although substantial intra-group heterogeneity remains. We also found higher SVR rates for personalised (100%; 95% CI, 97-100%) compared to stratified approaches (87.6%; 95% CI, 84.7-90.3%). The test for subgroup differences suggests that this effect is significant (p<0.001), although there were only four arms in the personalised strategy subgroup. Univariable meta-regression ( *Extended data*, Supplementary Table 3 ^10^) identified proportion of males, trial design, and duration as predictors of SVR at p<0.1. Duration ≤4 weeks retained significance in the multivariable model (coefficient, -0.4273480; p<0.001).

**Table 2.** Subgroup analysis - ‘maintain SVR’ group. Intention-to-treat SVR (%) estimates are presented alongside 95% confidence intervals (CI). Heterogeneity within subgroups was assessed using the I ^2^ test. P values represent the test for heterogeneity between subgroups

Subgroup	Arms (n)	Size (n)	SVR12	Lower CI	Upper CI	I ^2^ test	P value
**Ribavirin**							0.615
Yes	11	511	89.4	83.5	94.3	63.9	
No	25	5456	88.2	84.9	81.1	88.6	
**Duration**							**<0.001**
≤4 weeks	10	160	63.1	39.9	83.7	86.6	
6 weeks	14	383	81.1	75.1	86.6	46.6	
8 weeks	37	6235	94.2	92.3	95.9	79.4	
12 weeks	2	69	82.8	72.7	91.1	-	
**No. personalisation factors**							**0.008**
1	8	244	81.4	71.1	90.0	66.7	
2	42	2396	87.2	82.1	91.6	88.9	
≥3	13	4207	92.9	90.4	95.1	74.4	
**Optimisation strategy**							**<0.001**
Personalised	4	78	100.0	97.0	100.0	0.0	
Stratified	59	6769	87.6	84.7	90.3	87.6	
**DAA regimen**							0.502
Pangenotypic	17	452	86.8	78.9	93.2	76.0	
Genotype-specific	46	6395	89.0	85.8	91.8	88.5	
**Genotype**							0.891
Non-G3	57	6743	88.2	85.3	90.9	87.7	
G3	6	104	90.5	76.8	99.1	64.9	

### Improve SVR group meta-analysis

**Figure 3.**
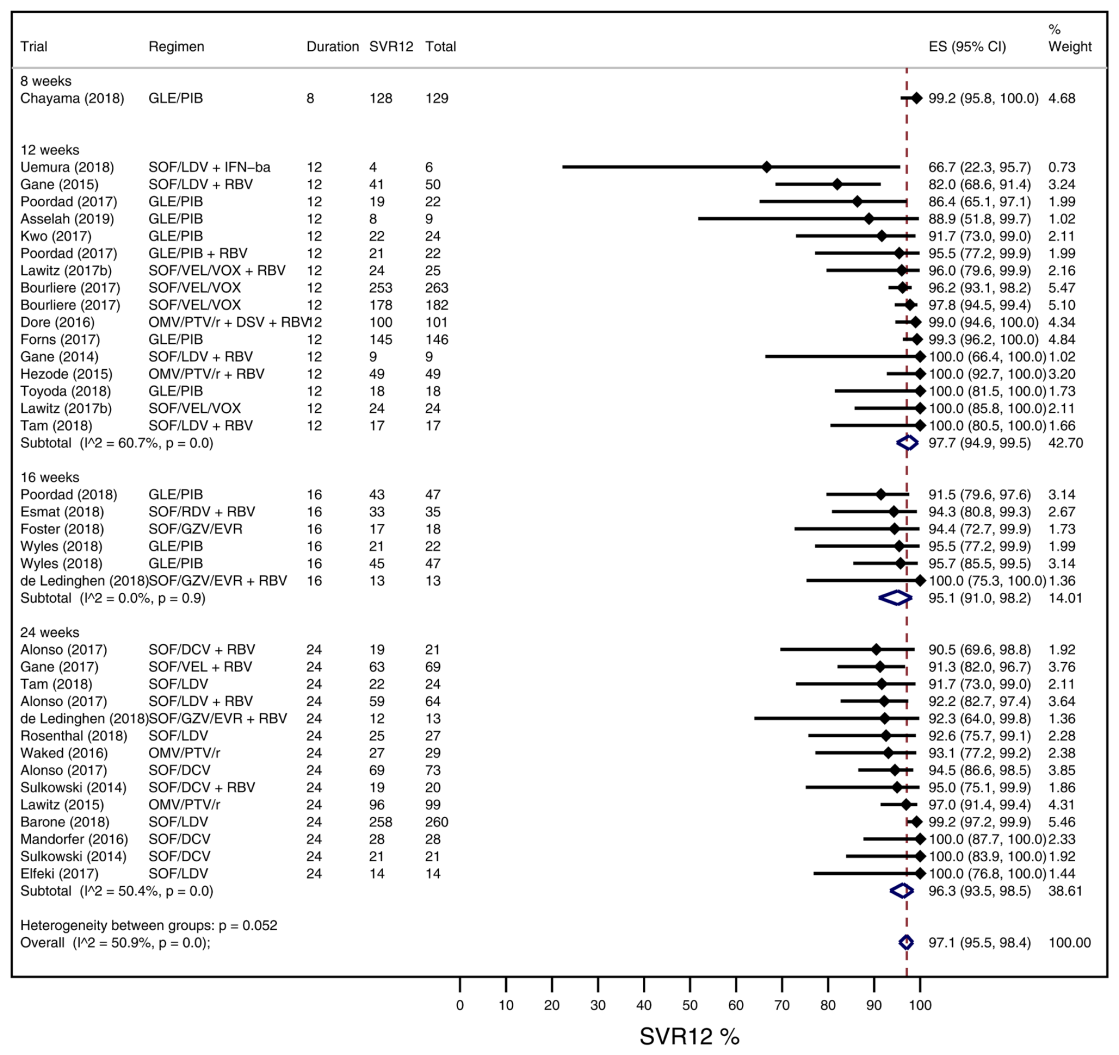
A Forest plot displaying pooled intention-to-treat SVR rates for optimised treatment arms in the improve SVR group stratified by treatment duration.

Figure 3 displays pooled intention-to-treat SVR rates for strategies that aim to improve SVR in the presence of negative predictors. Pooled SVR for 12 weeks duration was 97.7% (94.9-99.5%; I ^2^ = 60.7%), 16 weeks duration was 95.1% (91-98.2%; I ^2^ = 0%), and 24 weeks duration was 96.3% (93.5-98.5%; I ^2^ = 50.4%). There was no significant inter-group heterogeneity (p=0.052). The overall pooled SVR rate was 97.1% (95.5-98.4%; I ^2^ = 50.9%).

Subgroup analysis ( Table 3) found high pooled SVR rates (95.1-98%) in the presence of cirrhosis and/or prior treatment failure. Despite treatment optimisation, individuals with genotype 3 HCV suffer worse SVR rates (92.6%; 95% CI, 89.2-95.4%) versus non-genotype 3 (98.2%; 95% CI, 96.8-99.3%). The test for subgroup differences suggests that this effect is significant (p=0.001). Univariable meta-regression found no significant associations ( *Extended data*, Supplementary Table 4 ^10^). In subgroup analyses, additional RBV does not offer any advantage to the maintain SVR (p=0.615) or improve SVR group (p=0.243).

**Table 3.** Subgroup analysis - ‘improve SVR’ group. Intention-to-treat SVR (%) estimates are presented alongside 95% confidence intervals (CI). Heterogeneity within subgroups was assessed using the I ^2^ test. P values represent the test for heterogeneity between subgroups.

Subgroup	Arms (n)	Size (n)	SVR12	Lower CI	Upper CI	I2 test	P value
**Ribavirin**							0.243
Yes	14	508	96.0	92.5	98.6	49.2	
No	23	1532	97.7	96.1	99.0	47.1	
**Duration**							0.570 *
8 weeks *	1	129	99.2	95.8	100.0	-	
12 weeks	16	967	97.7	94.9	99.5	60.7	
16 weeks	6	182	95.1	91.0	98.2	0.0	
24 weeks	14	762	96.3	93.5	98.5	50.4	
**No. personalisation factors**							0.893
1	24	1732	96.8	94.9	98.3	60.0	
2	13	308	97.5	94.5	99.6	20.3	
**Risk groups**							0.273 *
Absent *	1	129	99.2	95.8	100.0	-	
Cirrhosis	11	761	98.0	95.5	99.7	52.8	
TE	19	990	96.4	93.8	98.5	53.6	
Cirrhosis + TE	6	160	95.1	90.6	98.4	0.0	
**Optimisation strategy**							0.763
Personalised	5	189	98.7	91.5	100.0	56.7	
Stratified	32	1851	96.7	95.1	98.1	50.0	
**DAA regimen**							0.591
Pangenotypic	20	1212	97.2	95.4	98.6	38.4	
Genotype-specific	17	828	96.8	93.7	99.1	62.3	
**Genotype**							**0.001**
Non-G3	29	1721	98.2	96.8	99.3	42.5	
G3	8	319	92.6	89.2	95.4	0.0	

*Note: for the subgroup analysis of treatment duration and clinical risk groups, the starred factors were excluded from the test for heterogeneity between subgroups due to n=1.

### Pooled analysis of resistance following treatment failure

Although short duration regimens resulted in lower SVR rates, there was a significantly lower prevalence of RASs at the point of treatment failure (p=0.0004 for trend) ( Table 4). However, data were only available for less than a third of treatment arms included in this study. A *post hoc* analysis stratified by RBV use did not find an association between RBV and RAS prevalence at treatment failure (data not shown).

**Table 4.** Prevalence of resistance-associated substitutions (RAS) at treatment failure. Analysis limited to treatment arms where sufficient data was available. Both enriched and emergent RASs are included. All classes of RASs are considered (NS3, NS5A, NS5B). Statistical comparison performed using the Chi-square test for trend in Prism v7 (GraphPad Prism, RRID:SCR_002798)

Duration (weeks)	Rx arms	RAS	No RAS	Total	%
4	2	7	4	11	63.6%
6	7	18	19	37	48.6%
8	8	23	19	42	54.8%
12	9	16	2	18	88.9%
16	3	7	0	7	100.0%
24	3	8	0	8	100.0%
Total	32	79	44	123	64.2%
**Chi-square test for trend p = 0.0004**					

Abbreviations: Rx – Treatment; RAS – resistance-associated substitution; % - proportion.

### Real-world studies reporting case-by-case personalisation

Four real-world studies described personalised therapy on a case-by-case basis, with treatment left to the physicians’ discretion. All personalised according to HCV treatment history, liver disease stage, and baseline RASs, with two also considering baseline HCV viral load. Rates of SVR were high using various combination DAA regimens (range, 94.7–100%).

### Assessment of quality and bias

Assessment of randomised studies using the Cochrane Risk of Bias tool found that the majority of studies are at a low risk of bias ( *Extended data*, Supplementary Figures 2 and 3 ^10^). Assessment of non-randomised studies using a modified Newcastle Ottawa Scale found that the majority of studies were of high quality ( *Extended data*, Supplementary Table 5 ^10^). However, no trials included an active comparator group.

## Discussion

The recent transformation in HCV treatment has led to increasingly widespread access to several IFN-free, DAA combinations. The WHO has set ambitious targets to eliminate HCV as a public health threat. Simplified universal treatments made possible by the new DAA therapies will be important in reaching such goals. However, a proportion of patients will not achieve cure with standard regimens. Furthermore, as countries approach elimination targets, the remaining pool of patients are likely to be harder to reach and/or harder to treat. For some patients, treatment optimisation may lead to better outcomes. The ideal balance between these approaches will vary in different settings.

This is the first detailed review of stratified and personalised treatment strategies in the combination DAA era. We identified a range of strategies, based on host and viral factors, with which the duration, combination, and/or dose of therapy may be adjusted to optimise rates of SVR. Such strategies are appropriate for a small but important proportion of hard-to-treat patients. Specialist infrastructure and expertise are often required, therefore these strategies are likely to be most relevant in well-resourced settings.

Individuals with HCV genotype 1a and NS5A RASs at baseline may experience worse outcomes when treated with standard durations of elbasvir/grazoprevir ^90^ or sofosbuvir/ledipasvir ^91^. The growing recognition of sub-genotypes that are relatively resistant to standard therapies (genotype 4r) ^92^ presents a challenge to simplified treatment strategies. A better understanding of regional sub-genotype differences and their impact on cure rates is required. High SVR rates (94.7-100%) from four real-world studies identified in this review support the use of baseline resistance testing within resistance-optimised treatment strategies. However, given the costs, technologies, and specialist input required, strategies based on resistance testing are likely to be confined to high-income settings at present.

Strategies for patients in whom treatment can be shortened are attractive, particularly where drug costs, adherence, or accessibility are a barrier ^93^. We report a pooled SVR of 94.2% (92.3-95.9%; I ^2^ = 79.4%) for 8 weeks’ therapy, where treatment was shortened from a 12 weeks standard of care. Where personalisation was based on simple clinical features alone (treatment history, liver disease stage), treatment durations <8 weeks result in a substantial reduction in SVR, especially for ≤4 weeks of therapy, and cannot currently be recommended.

High SVR rates are possible with ≤4 weeks of therapy using optimised strategies. Ovrehus *et al.* ^70^ added RBV and/or IFN to 4 weeks’ sofosbuvir/ledipasvir in treatment naïve, non-cirrhotic (F0-2), young (<50y), non-obese (BMI <30), injecting drug users with a low baseline HCV RNA (<2 million IU/mL). Intention-to-treat SVR rates were 93.8% (IFN + RBV; 100% per protocol) and 75% (RBV only; 93.2% per protocol). Lau *et al.* ^62^ added a protease inhibitor to 3 weeks’ sofosbuvir/daclatasvir or sofosbuvir/ledipasvir in genotype 1b non-cirrhotic individuals with a baseline HCV RNA <10 million IU/mL and an ultra-rapid virologic response (HCV RNA <500 IU/mL on day 2). They reported a 100% SVR rate. If we can accurately define those individuals in whom ≤4 weeks of therapy is appropriate, shortened strategies may become a realistic possibility. The significantly higher SVR rates with personalised (vs stratified) strategies and ≥3 host/viral optimisation factors found in our study, combined with the successful examples mentioned above, suggest that carefully optimised 4-week regimens may be possible.

Shortening treatment according to rapid virologic response (RVR) at week 4 was routine in the IFN era ^13^. However, almost all patients now achieve RVR by week 4 with DAA therapy ^94^. The optimum use of RGT has begun to be explored with modified RVR definitions. Lau *et al.* ^62^ applied ultra-rapid virologic response criteria (described above). Yakoot *et al.* ^88^ shortened sofosbuvir/daclatasvir to 8 weeks for genotype 4 if HCV RNA was undetectable at week 2. Both achieved high SVR rates (100% and 98.3%, respectively). Emerging research suggests RGT may be cost-effective in China ^95^. If RGT is to be a preferred strategy, more work is required to define a time point that is both predictive of treatment duration required to achieve cure and practical to implement.

Importantly, we provide evidence that the higher failure rates seen with short duration therapies are associated with a lower prevalence of resistance at the point of treatment failure relative to standard or extended duration therapies. Without individual participant data, we were unable to evaluate the influence of baseline RASs on short duration treatment therapies.

The recent European HCV treatment guidelines ^7^ emphasised RBV-free regimens. This avoids RBV-related toxicity and allows simplification of treatment algorithms. In general, evidence from this review does not support additional RBV when personalising treatment. An ongoing RCT is exploring biomarker-stratified short course (4–7 weeks) versus fixed duration (8 weeks) therapy and includes a factorial randomisation to RBV for each arm (see *Extended data*, Supplementary Table 6 for a summary of ongoing trials ^10^). This will hopefully address uncertainty regarding the utility of additional RBV for shortened duration therapy.

Despite stratified or personalised treatment strategies, we found significantly lower rates of SVR for individuals with genotype 3 HCV and poor prognostic factors (92.6% vs 98.2%; p=0.001). In the ASTRAL-3 study ^96^, 89% of treatment experienced, cirrhotic individuals with HCV genotype 3 achieved SVR following 12 weeks of sofosbuvir/velpatasvir. Higher SVR rates were reported with sofosbuvir/velpatasvir/voxilaprevir ^31^ and glecaprevir/pibrentasvir ^86^ in this difficult to treat group. Whilst simplified, decentralised care will be the cornerstone of treatment scale up, HCV genotype testing may yet be important in settings with a substantial genotype 3 prevalence. This will allow an appropriate regimen to be selected. In general, we find no evidence to support the extension of treatment beyond 12 weeks.

We identified one study that altered the dosing schedule. Uemura *et al*. ^83^ studied a 2-week lead-in period of daily IFN-beta injections before 12 weeks’ sofosbuvir/ledipasvir in DAA-experienced individuals. Only six participants were recruited (SVR 66.7%). Interferon-related toxicity and the poor response makes this an unattractive strategy. However, long-acting injectable DAA preparations, if developed, may allow reduced dosing schedules for difficult to access individuals.

We did not find any studies that used host genetics (e.g. IFN-lambda 4 (IFNL4)) to personalise treatment. There is evidence suggesting that an unfavourable IFNL4 genotype is associated with virologic relapse following short duration therapy with sofosbuvir/ledipasvir ^97^ and sofosbuvir/velpatasvir/voxilaprevir ^98^. This may be of relevance during the planning and analysis of future short duration trials.

There are limitations to this study. Firstly, we have meta-analysed heterogeneous studies that explore different DAA regimens, populations, and methodologies. We attempted to address this using a random effects model, which accounts for some heterogeneity. However, pooled SVR rates should be interpreted with caution. Secondly, we used a broad definition for stratified and personalised approaches to treatment optimisation. Consequently, we included several phase 2 exploratory studies that primarily aimed to establish optimum treatment duration. However, we feel that their inclusion provides interesting conceptual data. Thirdly, most data were derived from studies of sofosbuvir/ledipasvir. In the pangenotypic era the relative importance of sofosbuvir/ledipasvir may decrease. Fourthly, we have not included adverse events as an outcome for pooled analysis. Implementation of any optimised strategy will require confirmation of a similar or better side-effect profile compared to standard therapy. Finally, we have not considered cost-effectiveness. The cost of standard regimens varies widely and can be a barrier to access. In some settings, personalised regimens may be more cost-effective ^5^. However, the increased complexity associated with monitoring and infrastructure required for such strategies may present a pragmatic barrier.

Stratified and personalised treatment strategies have the potential to complement elimination efforts in some settings. Although existing standards of care for DAA therapy offer high SVR rates, there is evidence that treatment optimisation can improve outcomes in those with a higher predicted risk of failure. Whilst emerging data summarised in this review are encouraging, more evidence is needed to identify with confidence those individuals in whom SVR can be maintained whilst shortening treatment.

## Data availability

### Underlying data

All data underlying the results are available as part of the article and no additional source data are required.

### Extended data

Figshare: Data related to treatment optimisation for hepatitis C in the era of combination direct-acting antiviral therapy: systematic review and meta-analysis. https://doi.org/10.6084/m9.figshare.9636983.v1 ^10^.

This project contains the following extended data:

- Supplementary figure 1: Thematic map exploring strategies for treatment optimisation. The duration, combination and/or dose of a treatment regimen is optimised for the individual receiving therapy. Abbreviations: RBV - ribavirin; DAA - direct-acting antiviral; IFN - interferon.
- Supplementary figure 2: Cochrane risk of bias tool for randomised controlled trials summary - review authors' judgements about each risk of bias item for each included study. Prepared using Review Manager v5.3 (RevMan, RRID:SCR_003581).
- Supplementary figure 3: Risk of bias graph - review authors' judgements about each risk of bias item presented as percentages across all included studies. Prepared using Review Manager v5.3 (RevMan, RRID:SCR_003581).
- Supplementary table 1: Summary of Ovid search strategy - Medline and Embase. Last conducted on 4 ^th^ July 2019.
- Supplementary table 2: Individual study characteristics. The factors used for stratification or personalisation, treatment strategy adopted, and resultant SVR rates (intention-to-treat and per protocol) are presented for each treatment arm.
- Supplementary table 3: Meta-regression - ‘maintain SVR group’. Clinical and methodological variables were subject to univariable random effects meta-regression. Only those variables with p≤0·1 on univariable analysis were carried forward to the multivariable model. Significance of variables in multivariable model taken at p≤0·05 level. Upper (UCI) and lower (LCI) 95% confidence intervals are presented.
- Supplementary table 4: Meta-regression - improve SVR group. Clinical and methodological variables were subject to univariable random effects meta-regression. There were no significant associations and therefore a multivariable model was not constructed. Upper (UCI) and lower (LCI) 95% confidence intervals are presented.
- Supplementary table 5: Modified Newcastle Ottawa Scale for quality assessment of nonrandomised studies. In this modified scale, a study can be awarded a maximum of one star for each item within the Selection and Outcome categories. The comparability domain was removed to account for the non-comparative nature of included studies (unmodified version can be found at: http://www.ohri.ca/programs/clinical_epidemiology/nosgen.pdf).
- Supplementary table 6: Ongoing randomised controlled trials that are evaluating stratified or personalised treatment strategies.
- Excel data sheet: The data were extracted into a pre-designed Excel template and this was subsequently imported into Stata for the meta-analysis. This sheet contains all extracted data for each treatment arm.
- Stata .do file: The .do file used to perform the meta-analysis in Stata. All commands and packages used are included within this file.

### Reporting guidelines

Figshare: PRISMA checklist for ‘Treatment optimisation for hepatitis C in the era of combination direct-acting antiviral therapy: a systematic review and meta-analysis’. https://doi.org/10.6084/m9.figshare.9636983.v1 ^10^.
