# Supplementary material for: Treatment optimisation for hepatitis C in the era of combination direct-acting antiviral therapy: a systematic review and meta-analysis
Source: Wellcome Open Res. 2019 Sep 6;4:132. [Version 1] doi: 10.12688/wellcomeopenres.15411.1 (PMC6854875; doi:10.12688/wellcomeopenres.15411.1)
Supplement: Peer review history

# Peer review: Reviewer response for version 1

**DOI:** 10.21956/wellcomeopenres.16849.r36494

**Published:** 2019-11-13

**Authors:** Anne Lindebo Holm Øvrehus

**Affiliations:** 1 Department of Infectious Diseases, Odense University Hospital, Odense, Denmark

This paper presents a systematic review and meta-analysis on available data on chronic hepatitis treatment with direct acting antivirals from trials aiming at either shortening treatment "optimization" or enhancing response "maintaining" in difficult patients. The review thus includes both industry initiated phase 2 and 3 trials and investigator initiated more experimental trials. The authors have made an impressive effort and an important contribution in setting up a conceptual framework for the analysis and communication of studies on treatment optimization in Chronic Hepatitis C in the DAA era. The decision to make separate analysis for studies aiming at maintaining SVR in easy to treat and optimizing SVR in hard to treat patients is just. It might however have been a better strategy to make separate papers to overcome the large conceptual span of these somewhat opposing aims. That said the benefit of being able to compare RAS between the two strategies justifies this approach.

 The paper comprises a wealth of information and although the method is mainly that of a systemic review, the discussion is more based on the narrative or concept.

**Aim**

 The stated aim is to initiate a discussion on the role of treatment optimization and in this the authors have succeeded.

**Method**

 Well described and choices made in the process are supported.

 Please define "enriched" RAS (does it mean RAS present at baseline but sequence population proportion had increased at failure?).

**Results**

 Table 1

 Given that data are analyzed separately for the "Maintain" and "Optimize" studies (and that DAA used in the two groups are quite different) pooling them into one table is a little misleading.

 Table 4

 Important point - Please consider if presenting data on treatment emergent RAS and "enriched" RAS should be separate. 

 Treatment emergent RAS is a major concern in treatment failure patients and it would be interesting to see these data.

**Discussion**

 Opens with a focus on hard to treat patients and optimizing SVR rates.

 As the results sections starts with the maintain SVR in absence of negative predictors approach it is a little confusing.

 That the majority of studies were conducted using LDV/SOF is rightly stated as a limitation, as is that safety was not included as a factor. 

 The important finding that short therapy at 4 weeks does not lead to development of RAS is an important finding in a public health perspective (fear of initiating therapy in vulnerable populations that might discontinue early) but the actual results are not mentioned.

 Genotype 4r is singularly mentioned as genotype relatively resistant to standard DAA - there are many others - so maybe better to leave out?

 The discussion does not explicitly include consideration on the target population demographic characteristics for treatment optimization. Would in general be considered to be populations where adhering to an extended course of treatment can be challenging - like in very active PWID or limited resource settings?

I confirm that I have read this submission and believe that I have an appropriate level of expertise to confirm that it is of an acceptable scientific standard.

# Peer review: Reviewer response for version 1

**DOI:** 10.21956/wellcomeopenres.16849.r36505

**Published:** 2019-10-03

**Authors:** Kali Zhou

**Affiliations:** 1 Department of Medicine, Division of GI/Liver, University of California, San Francisco, CA, USA

This is a well-written and comprehensive systematic review and meta-analysis (with meta-regression) on treatment optimization for hepatitis C using direct-acting agents. This is an interesting topic and relevant as we push the boundaries of simplifying and improving HCV regimens for patients to ultimately decrease costs, improve adherence, and make elimination a reality. The majority of the significant findings are not surprising and have mostly been adopted as SOC. I commend the authors for completing this review.

 The methodology was fairly complex, with several layers, but overall well-defined. The first is separation of predictor (optimization strategy) to stratified strategy (defined as subgroups related to prognostic factors such as severity of liver disease and prior treatment history) and personalized strategy (individual host and viral factors such as viral resistance). There were also two goals of treatment optimization that were separated in analysis: 1) improve SVR in those with negative prognostic factors and 2) maintain current SVR rates with easier or shorter regimens in those without negative prognostic factors. Each optimization strategy could have been done by changing duration, drug or both. Primary outcome of meta-analysis was % SVR. The purpose of meta-regression was to identify clinical and methodological contributors to heterogeneity in findings.

 The authors were thorough in their review methodology and adhered to PRISMA guidelines. Selection of studies was performed by one reviewer while a 2 ^nd^ reviewer screened 20% of studies, reasonable given their initial search identified >8000 articles. However, only one reviewer performed data extraction and having a minimum of 2 is standard.

 Reasons for exclusion in Figure 1 were reasonable. It is surprising that no additional records were identified (perhaps in reference lists?) although the broad initial search likely limited missed publications. I would consider excluding studies using IFN in meta-analysis due to anticipated heterogeneity (Uemura).

 Bias was assessed with separate tools for RCTs and observational studies. Publication bias was not assessed although the authors could have considered Peter’s test, which can be used with proportions.

 The major finding of the study is that shortened duration of therapy (8 weeks) is an acceptable strategy to maintain SVR in those with no negative prognostic factors with pooled SVR of 94%, but SVR drops with 6 and 4 week regimens. The authors provide subgroup analysis by addition of RBV, number of personalization factors, strategy, regimen, and GT – however, what specific personalization factors were considered is not clearly evident for these patients with no negative prognostic factors (presumably, stage of disease and prior treatment history are not applicable).

 For improving SVR in those with negative prognostic factors, duration did not significantly impact SVR (thus 12 as good as 24). In subgroup analysis, GT3 patients (not unexpected) had lower SVR rates. I would be interested in differences between arms with SVR<95 and >95% using duration of 12 weeks and hypothesized reasons for lower SVR reports in these arms of smaller sample size. Also, did SVR rates for 12-week regimens differ by negative prognostic factor (not so overall but not evaluated by duration). At present time, however, since 12-week regimens are standard of care for negative prognostic groups, this issue is not major.

 The authors did not evaluate effect of optimized treatment type (duration and duration+combination as two largest subgroups as shown in Table 1).

 The renal failure subpopulation was not discussed (or explicitly excluded) but I am curious if any studies identified attempted to optimize treatment in this group.

I confirm that I have read this submission and believe that I have an appropriate level of expertise to confirm that it is of an acceptable scientific standard.
